# The effects of the dental methacrylates TEGDMA, Bis‐GMA, and UDMA on neutrophils in vitro

**DOI:** 10.1002/cre2.296

**Published:** 2020-06-16

**Authors:** Sara Alizadehgharib, Ann‐Karin Östberg, Agnes Dahlstrand Rudin, Ulf Dahlgren, Karin Christenson

**Affiliations:** ^1^ Department of Oral Microbiology and Immunology Institute of Odontology, The Sahlgrenska Academy, University of Gothenburg Gothenburg Sweden

**Keywords:** dentin‐bonding agents, methacrylates, neutrophil extracellular traps, neutrophils

## Abstract

**Objectives:**

The prevalent usage of methacrylates in modern dentistry demands good knowledge of their biological impacts. While there have been several studies demonstrating the effects of different methacrylic monomers on mononuclear white blood cells, very little is known about the effects caused by these monomers on neutrophilic granulocytes. The objective of this study was to add novel knowledge about how neutrophils are affected by exposure to triethylene glycol dimethacrylate (TEGDMA), urethane dimethacrylate (UDMA), and bisphenol A glycol dimethacrylate (Bis‐GMA) alone or in combinations.

**Materials and Methods:**

Isolated neutrophils were cultured in the presence or absence of methacrylates. The IL‐8 release was measured using a DuoSet ELISA development kit. Apoptosis and necrosis were analyzed using flow cytometry. The formation of neutrophil extracellular traps (NETs) was investigated using Sytox green DNA staining combined with microscopically examination of released DNA and myeloperoxidase (MPO).

**Results:**

The release of IL‐8 was significantly increased after exposure to TEGDMA, Bis‐GMA, UDMA, or TEGDMA in combination with Bis‐GMA or UDMA compared to the unstimulated controls. Exposure to TEGDMA, UDMA, and Bis‐GMA for 24 hr separately or in combination did not affect apoptosis or necrosis of the exposed neutrophils. NET structures were formed by neutrophils after exposure to the different combinations of the methacrylates.

**Conclusion:**

The combination of TEGDMA and Bis‐GMA had a synergistic proinflammatory effect on neutrophils by increasing the release of IL‐8 and the formation of NET structures. The changes in the normal functions of neutrophils caused by methacrylate exposure may lead to altered inflammatory response and relate to previously reported adverse immune reactions caused by these substances.

## 1 INTRODUCTION

Dental acrylate and methacrylate monomers are used in different polymer resin‐based materials (PRMs) (Aalto‐Korte, Alanko, Kuuliala, & Jolanki, 2007; Nocca et al., 2014; Schmalz, Krifka, & Schweikl, 2011; Van Landuyt et al., 2011). The most widely used monomers for the preparations of dental resins are crosslinking dimethacrylates such as triethylene glycol dimethacrylate (TEGDMA), bisphenol A glycol dimethacrylate (Bis‐GMA), urethane dimethacrylate (UDMA), and bisphenol A ethoxylated dimethacrylate (Bis‐EMA). Various mixtures of these monomers are used to achieve desired mechanical and physical properties together with reinforcement materials (e.g., fillers). Although relatively few clinical adverse effects have been reported in patients and dental personnel regarding these materials, side effects occur. Reactions such as irritation of the eyes, skin, and mucous membranes as well as contact dermatitis (with a prevalence of ≈1%), stomatitis, asthma, liver toxicity, fertility disturbances, and disturbances of the central nervous system have been reported (Fisher, 1954; Giunta, Grauer, & Zablotsky, 1979; Gosavi, Gosavi, & Alla, 2010; Kiec‐Swierczynska, 1996; Lindstrom, Alanko, Keskinen, & Kanerva, 2002; Venables, Narayana, & Johnston, 2016; Wallenhammar et al., 2000; Wrangsjo, Swartling, & Meding, 2001). Patients are exposed to methacrylate monomers when they receive restorations, which release residual monomers for months after the restoration has been placed (Ferracane, 1994; Ortengren, Wellendorf, Karlsson, & Ruyter, 2001). However, the personnel in dental clinics are usually exposed to these substances on a daily basis and are at higher risk of adverse effects.

A multitude of immunomodulatory effects of different acrylates and methacrylates have previously been reported (Aalto‐Korte et al., 2007; Alizadehgharib, Östberg, & Dahlgren, 2017a, 2017b; Andersson & Dahlgren, 2008, 2010, 2011; Samuelsen, Dahl, Karlsson, Morisbak, & Becher, 2007; Sandberg, Bergenholtz, Kahu, & Dahlgren, 2005; Sandberg & Dahlgren, 2006; Sandberg, Kahu, & Dahlgren, 2005). Thus, these substances disturb the delicate homeostasis of the immune system. Some of the effects on the immune system are changes in production of cytokines which may impact initiation and containment of inflammation (Alizadehgharib et al., 2017a; Golz et al., 2016; Gregson, Terrence O'Neill, Platt, & Jack Windsor, 2008; Moharamzadeh, Brook, Scutt, Thornhill, & Van Noort, 2008; Moharamzadeh, Franklin, Brook, & van Noort, 2009; Schmalz, Schweikl, & Hiller, 2000). These monomers have also been described to have adjuvant properties (Alizadehgharib et al., 2017a; Bando et al., 2014; Sandberg, Kahu, et al., 2005) which among other effects caused increased proliferation of splenocytes in vivo (Andersson & Dahlgren, 2011).

While there have been several studies demonstrating the effects of 2‐hydroxyethyl methacrylate (HEMA) and TEGDMA on the immune system, very little is known about the effects caused by other methacrylates such as Bis‐GMA and UDMA. The previous studies have investigated the effects of exposure to each monomer separately, while both patients and dental personnel are usually exposed to materials containing a combination of different monomers. Furthermore, most of the previously published research have studied the effects of methacrylates on mononuclear white blood cells (Alizadehgharib et al., 2017a, 2017b; Bolling et al., 2013; Golz et al., 2016; Grande et al., 2015; Heil, Volkmann, Wataha, & Lockwood, 2002; Krifka et al., 2010), while the effects of these materials on the polymorphonuclear neutrophils (PMN) are still unknown.

Neutrophil granulocytes are very potent phagocytes that swiftly transmigrate from blood to surrounding tissue upon signals of microbial invasion or endogenous damage. Neutrophils express a variety of pattern recognition receptors and are therefore very responsive to all types of danger signals. Once the neutrophils arrive to an infected site, they eliminate microorganisms and cell debris, by phagocytosis, that is, ingestion, killing, and degradation of the prey inside special cell compartments. Neutrophils contain a multitude of toxic substances and enzymes which are used to kill microbes and, if released extracellularly, may damage the surrounding tissues. To prevent tissue damage by release of toxic substances, neutrophils are programmed to die by apoptosis, a process that do not release inflammatogenic agents, and they are then cleared from the site by other phagocytes. The apoptotic process and the subsequent clearance of the dead neutrophils are of utter important, as it is necessary for dampening and resolution of the inflammation (Greenlee‐Wacker, 2016). However, under certain circumstances, for example, if the prey escapes engulfment due to size or shape, neutrophils can enter a more violent, pro‐inflammatory type of death called NETosis. In this process, neutrophil extracellular traps (NETs) are formed, that is, the DNA, together with granule content (e.g., myeloperoxidase (MPO) and serine proteases) are thrown out from the cell to the extracellular milieu. The NETs can capture pathogens, but NET formation is also described in several noninfectious diseases as cancer, rheumatoid arthritis, gout, and systemic lupus erythematosus as reviewed by Papayannopoulos (Papayannopoulos, 2018) or in contact with synthetic chemicals (Guimaraes‐Costa, Nascimento, Wardini, Pinto‐da‐Silva, & Saraiva, 2012).

The purpose of this study was to add novel knowledge about the effects of the methacrylates TEGDMA, UDMA, or/and Bis‐GMA on neutrophil granulocytes.

## 2 MATERIALS AND METHODS

### 2.1 Isolation of human neutrophils

Fresh blood cells from healthy blood donors were obtained from Sahlgrenska University Hospital in Gothenburg, Sweden. Peripheral blood neutrophil isolation was performed as first described by Boyum (Boyum, 1968). Briefly, after removal of red blood cells in a sedimentation step, the suspension was centrifuged on Ficoll‐Paque Plus (GE Healthcare Bio‐Sciences, Uppsala, Sweden). The pelleted neutrophils were collected and the remaining erythrocytes were lyzed by hypotonic treatment. All the washing of the neutrophils was performed using Krebs Ringer Phosphate buffer (KRG) and in the end, the isolated neutrophils were suspended in KRG supplemented with Ca^2+^ (1 mM) and kept on ice until the assays were performed (within less than 1 hr).

### 2.2 Overnight incubation of neutrophils

Briefly, 450 μl isolated human neutrophils (5 × 10^6^ cells/ml) were suspended in RPMI complemented with 10% FCS and 1% PEST and incubated for 30 min at 37°C in the presence of 5% CO_2_. Next, 50 μl of the methacrylates (dissolved in DMSO, 500 μM), alone or in combinations (Table 1), were added to the neutrophils. For controls, buffer, proapoptotic anti‐CD95 antibody (10 μg/ml, BioLegend) or antiapoptotic LPS (*Escherichia coli* (serotype O127:B8) lipopolysaccharide (LPS), 100 ng/ml, Sigma) were used (Christenson, Bjorkman, Tangemo, & Bylund, 2008; Park, Jin, Song, Park, & Kwak, 2007; Ren et al., 2008) and the neutrophils were further incubated overnight (for 20 hr).

**TABLE 1:** The different combinations of the methacrylates used in the study

Methacrylate	TEGDMA	Bis‐GMA	UDMA	Bis‐GMA + TEGDMA	UDMA + TEGDMA
Concentration	500 μM	500 μM	500 μM	500 μM + 500 μM	500 μM + 500 μM

The next day the neutrophils were evaluated for cell death and the cell‐free supernatants were saved for evaluation of IL‐8 content as described below.

### 2.3 Cytokine assay

After 20 hr incubation of the neutrophils (described above), 200 μl cell free supernatant was carefully aspirated from each sample and used for cytokine analysis. For this purpose, a DuoSet ELISA Development kit from R&D Systems (Abingdon, UK) was used according to the manufacturer's instructions.

### 2.4 Evaluation of cell death using FACS analyses

After removal of the cell free supernatant (as described above) the undisturbed cell pellets were suspended in the culture media and 200 μl of the neutrophils from each cultured sample was collected and washed in 2 ml Annexin buffer (1 mM Hepes, 14 mM NaCl, 0.25 mM CaCl_2_, [pH 7.4]). The cell pellets were thereafter resuspended in 100 μl Annexin buffer, supplemented with 2 μl Annexin V‐FLUOS and 5 μl 7‐AAD and incubated for 10 min in the dark. Thereafter, another 300 μl Annexin buffer was added, and samples were subjected to FACS analysis using an Accuri C6 (Becton Dickinson, Mountain View, CA, USA). At least 10,000 events were acquired, and neutrophils were gated on the basis of side and forward scatters. Apoptosis (Annexin‐V+/7‐AAD‐) was assessed on the basis of Annexin V‐FLUOS fluorescence, as measured in the fluorescence 1 (FL1) channel, and necrosis (Annexin‐V+/7‐AAD+) was assessed on the basis of membrane permeability to 7‐AAD, as measured in the FL3 channel.

All data were analyzed using CFlow and GraphPad Prism software.

### 2.5 Measurement of extracellular DNA with Sytox green

Sytox Green DNA stain (Thermo Fisher Scientific, Gothenburg, Sweden) is a membrane impermeable dye that can be used to measure extracellular DNA from ruptured cells (Gupta, Chan, Zaal, & Kaplan, 2018). As extracellular DNA release is a marker of NET generation, Sytox green has, in combination with other methods, for example, microscopy, been described as a tool to evaluate NETosis (Bjornsdottir et al., 2017; de Buhr & von Kockritz‐Blickwede, 2016; Kraaij et al., 2016; Van, Alvarez de Haro, Bron, & Desbois, 2020; van Breda et al., 2019).

For the assay, neutrophils in RPMI (without phenol red: Thermo Fisher Scientific) were added to a black 96‐well plate at a concentration of 0.5 × 10^5^ cells/well together with the Sytox Green DNA stain (2.5 μM; Molecular Probes).

Different combinations of the methacrylates at a concentration of 500 μM were incubated with cells and DNA stain at 37°C and 5% CO_2_. Phorbol 12‐myristate 13‐acetate (PMA; Sigma‐Aldrich) at a concentration of 50 nM/well and 1% Triton X‐100 were used as positive controls. Sytox Green fluorescence was measured after 0, 1, 2 and 3 hr of incubation at 485/535 nm in a CLARIOstar plate reader (BMG Labtech).

### 2.6 Microscopic visualization of NETosis

Isolated human neutrophils (5.5 × 10^5^ cells/ml) were suspended in RPMI and added to poly‐lysine‐coated 24 well glass bottom plates (Cellvis, California, USA) and incubated at 37°C in the presence of 5% CO_2_ for 10 min. After stimulation with PMA (50 nM) or the methacrylates, the cells were further incubated at 37°C in the presence of 5% CO_2_ for 3 hr (the incubation time was chosen from the results received after Sytox Green DNA stain). Cells were fixed in 4% paraformaldehyde for 30 min at room temperature and, permeabilized with cold acetone and methanol (1:1) for 5 min. To visualize NETs, the samples were stained with antibodies against myeloperoxiadase (MPO, DAKO), followed by secondary antibody staining (Donkey Anti‐Rabbit IgG H&L Alexa Fluor® 488 purchased from ThermoFisher). Finally, the coverslips were mounted with ProLong™ Gold antifade mountant with the DNA stain DAPI (ThermoFisher). The cells were visualized using an Olympus BX41 epifluorescent microscope with the CellSens software.

### 2.7 Statistical analysis

All analyses were performed using GraphPad Prism (GraphPad Software Inc., San Diego, CA, USA). For all tests, a *p*‐value of <.05 was considered statistically significant, *p < .05, **p < .01 and ***p < .001. Statistical comparisons between paired samples were made using the Wilcoxon matched‐pairs signed‐rank test.

## 3 RESULTS

### 3.1 Release of IL‐8

**FIGURE 1:**
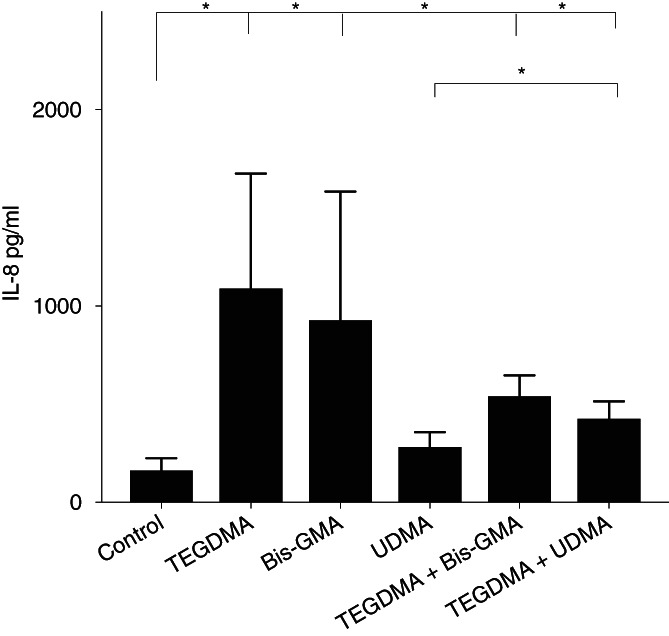
Production of IL‐8 in human neutrophils. Neutrophils were exposed to methacrylates (500 μM) alone or different combinations of triethylene glycol dimethacrylate (TEGDMA), bisphenol A glycol dimethacrylate (Bis‐GMA) and UDMA for 20 hr and incubated at 37°C. Total IL‐8 content in the cultures were measured by ELISA and is shown as mean + *SEM*

To study IL‐8 release after exposure to the different methacrylates, culture supernatants were collected after overnight incubation and the concentration of IL‐8 was determined. The concentration of IL‐8 was significantly higher in the supernatants of cultures exposed to TEGDMA (*p* = .0312), Bis‐GMA (*p* = .0312), and TEGDMA in combination with Bis‐GMA (*p* = .0156) and TEGDMA in combination with UDMA (*p* = .0156) compared to the supernatants of cultures of unexposed cells (Figure 1).

### 3.2 FACS analyses of cell death

**FIGURE 2:**
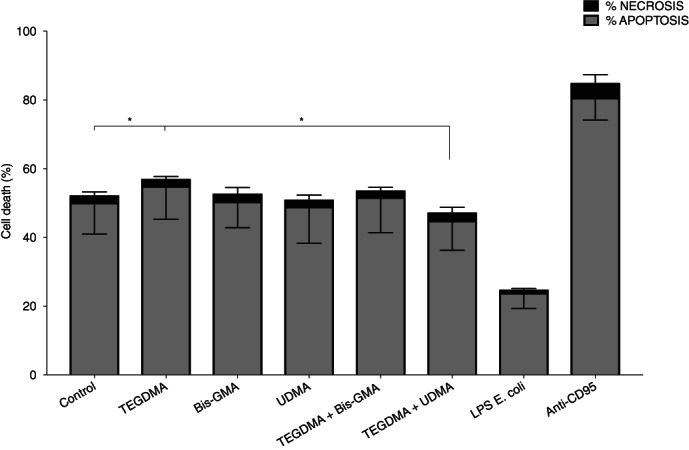
FACS analyses of cell death. Neutrophils were exposed to the pro‐ (anti‐CD95 antibody, 10 μg/ml) and antiapoptotic (lipopolysaccharide (LPS), 100 ng/ml) controls or the methacrylates (500 μM) alone or different combinations of triethylene glycol dimethacrylate (TEGDMA), bisphenol A glycol dimethacrylate (Bis‐GMA) and UDMA for 20 hr and incubated at 37°C. The cell death of the neutrophils was evaluated by flow cytometry. Apoptotic cells labeled with Annexin V are presented as grey parts of the bars, while necrotic cells positive for both Annexin V and 7‐AAD are seen as black parts of the bars. Results are from six independent experiments shown as mean ± *SD*

Neutrophils are short‐lived cells that die by apoptosis within a couple of days. The apoptotic process can be increased or delayed depending on surrounding factors in an inflammatory milieu. In concordance with previous studies, after 20 hr incubation approximately 50% of the neutrophils where viable (Annexin V−/7‐AAD–) and around 50% were apoptotic (Annexin V+/7‐AAD–) in the absence of stimulation (Christenson, Thoren, & Bylund, 2012; Dubey et al., 2016; Hosseinzadeh, Messer, & Urban, 2012). Necrosis (Annexin V+/7‐AAD+) was consistently below 5%. Small variations in the apoptosis and necrosis of the neutrophils were observed after exposure to the different methacrylates alone or in combinations. The total cell death was significantly lower when cells were exposed to UDMA and TEGDMA in combination, compared to TEGDMA alone (*p* = .0017) (Figure 2).

### 3.3 Cytotoxicity/DNA release measurements with Sytox green DNA stain

**FIGURE 3:**
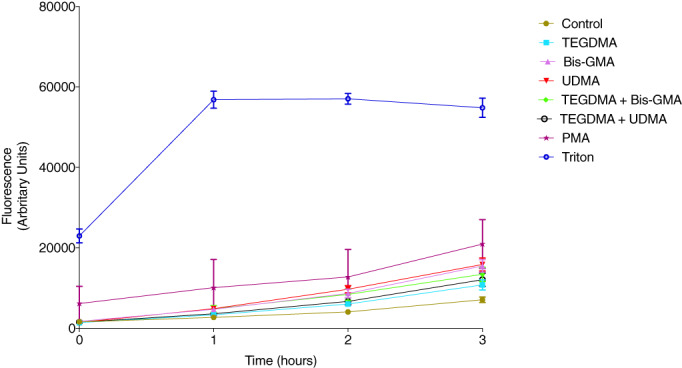
Methacrylates induce Sytox green fluorescence in neutrophils. Sytox Green fluorescence measured from neutrophils incubated with or without methacrylates (500 μM) alone or in different combinations of triethylene glycol dimethacrylate (TEGDMA), bisphenol A glycol dimethacrylate (Bis‐GMA) and UDMA for 0, 1, 2, and 3 hr. Phorbol myristate acetate (PMA, 50 nM) and Triton X‐100 (TX‐100; 1%) were used as controls. Results are from seven independent experiments shown as mean ± *SD*

**FIGURE 4:**
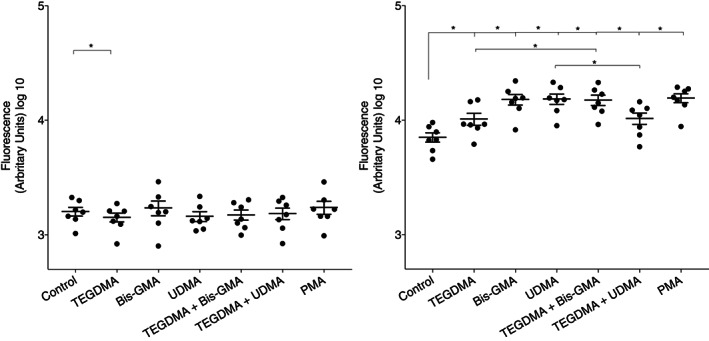
Measurements of cytotoxicity/DNA release with Sytox Green DNA stain. The Sytox Green fluorescence was measured from neutrophils (*n* = 7) incubated with or without 500 μM of methacrylates alone or in different combinations of triethylene glycol dimethacrylate (TEGDMA), bisphenol A glycol dimethacrylate (Bis‐GMA) and UDMA (500 μm) for 3 hr incubated at 37°C. Phorbol myristate acetate (PMA) was used as a positive control. Results from seven independent experiments are shown at time 0 hr (left) and 3 hr (right) as mean ± *SEM*

In a way to evaluate NETosis, a cell impermeable DNA binding Sytox Green dye was used to measure release of DNA to the extracellular environment after cell exposure to different combinations of the methacrylates. Monitoring Sytox green fluorescence every hour for 3 hr showed that exposure to the methacrylates increased the amount of neutrophil released extracellular DNA (Figure 3). After 3 hr of incubation, significantly increased amounts of DNA were observed when cells were exposed to TEGDMA (*p* = .0156), Bis‐GMA (*p* = .0026), UDMA (*p* = .0043), and Bis‐GMA in combination with TEGDMA (*p* = .0043), compared to unexposed cells. The combination of UDMA and TEGDMA also resulted in significantly increased DNA release (*p* = .0156) (Figure 4). Cell exposure to TEGDMA in combination with Bis‐GMA showed significantly higher DNA release compared to TEGDMA alone (*p* = .0312). Exposure to UDMA alone showed significantly higher release of DNA compared to UDMA in combination with TEGDMA (*p* = .0156).

**FIGURE 5:**
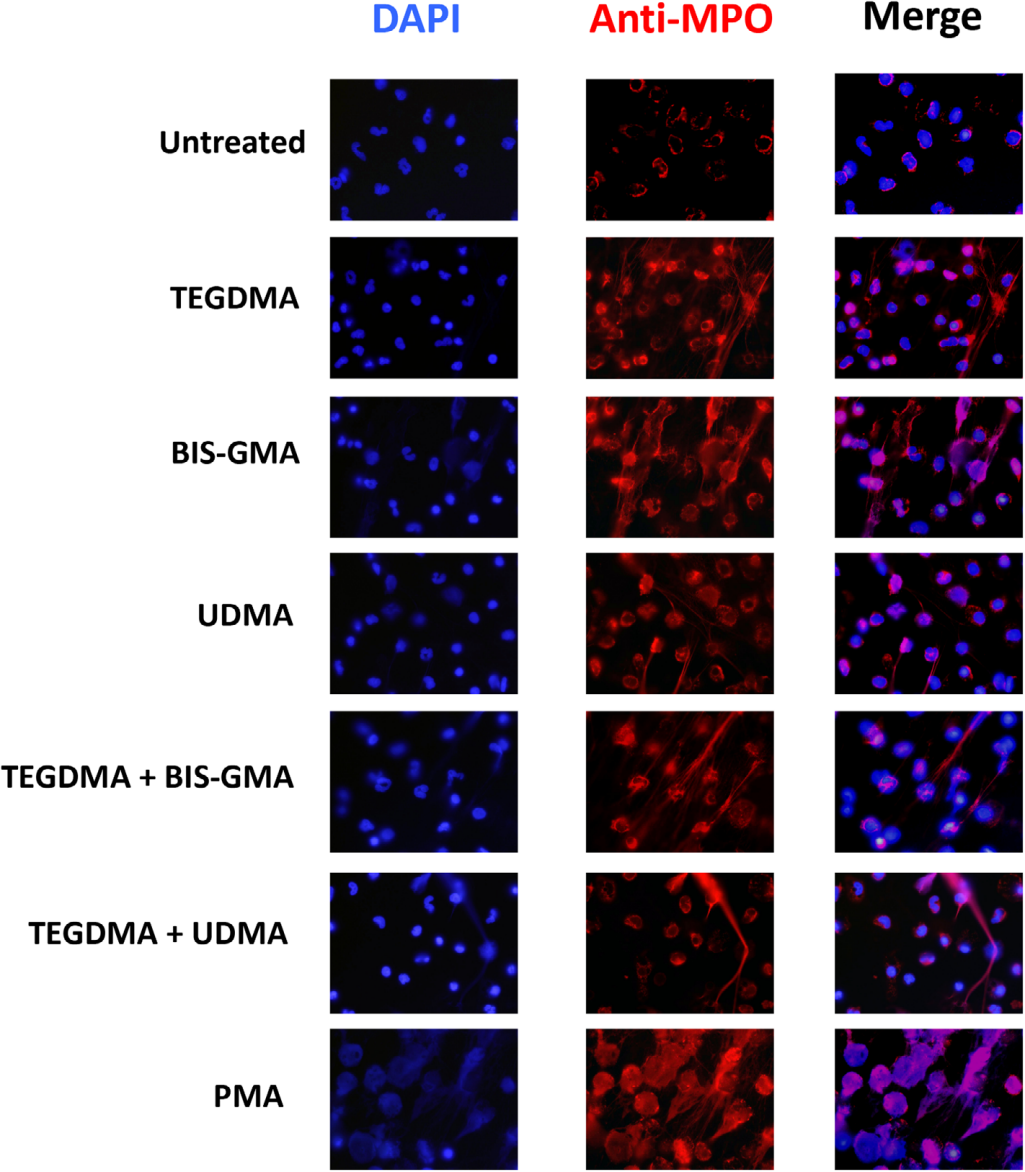
DNA release measurements with immunofluorescence microscopy. Neutrophils were exposed to phorbol myristate acetate (PMA, 50 nM) or the methacrylates (500 μM) alone or in different combinations of triethylene glycol dimethacrylate (TEGDMA), bisphenol A glycol dimethacrylate (Bis‐GMA) and UDMA for 3 hr incubated at 37°C. The cells were fixed and stained for DNA (blue) and MPO (red). The images are from one representative experiment out of two independent experiments performed. The cells were visualized using an epifluorescence microscope

These findings show that the investigated monomers affected the neutrophils to release extracellular DNA, and to further evaluate if this were due to NETosis, the assay were confirmed by immunofluorescence (Figure 5). NETs consist of DNA clad with proteins from the various granules, for example, neutrophil elastase, cathepsin G, and MPO from azurophilic granules, lactoferrin from specific granules, and gelatinase from the gelatinase granules. In Figure 5, DNA is stained blue using DAPI while the MPO is shown in red.

## 4 DISCUSSION

Resin‐based composite materials mainly consist of fillers embedded in a polymer resin‐based matrix (Peutzfeldt, 1997) that comprises of different elements, such as methacrylate monomers and additives (Schmalz & Arenholt‐Bindslev, 2009). The most extensively used monomers for the preparations of dental resins are crosslinking dimethacrylates such as TEGDMA, Bis‐GMA, UDMA, and bisphenol A ethoxylated dimethacrylate (Bis‐EMA). Free residual monomers (≈3%/volume of the matrix) are present in the dental restorations and can be released into the oral cavity mostly within hours after placement of the restoration (Ferracane, 1994). Elution of residual monomers may, however, continue for several days or for months depending on the degree of conversion, cross‐linkage of the matrix, and the monomer size and wear (Aalto‐Korte et al., 2007; Bindslev & Schmalz, 2009; Carol Dixon Hatrick, 2016; Ortengren et al., 2001; Schmalz et al., 2011).

It has previously been demonstrated that acrylate/methacrylate monomers may cause a wide range of adverse health effects. Biological adverse reactions can take place either locally or distant from the site of contact (i.e., systemically). Therefore, it is important to have a holistic view of the effects of biomaterials on the immune system and not only concentrate on the local oral environment. This knowledge is necessary for better understanding and for the ability to explain clinical characteristics, provide potential therapeutic strategies and to prevent unwanted health effects in an accurate way. Previous studies have reported many immunomodulatory effects of different acrylates and methacrylates (Aalto‐Korte et al., 2007; Alizadehgharib et al., 2017a, 2017b; Alizadehgharib, Östberg, Larsson, & Dahlgren, 2018; Andersson & Dahlgren, 2008, 2010, 2011; Samuelsen et al., 2007; Sandberg, Bergenholtz, et al., 2005; Sandberg & Dahlgren, 2006; Sandberg, Kahu, et al., 2005; Östberg, Alizadehgharib, & Dahlgren, nd). However, the in vitro studies have mainly been conducted on mononuclear white blood cells, while the effects on neutrophils are still unknown. As neutrophils always are present in the oral cavity and very responsive to all types of danger signals, the effects of methacrylates on these cells are of great interest. The methacrylates are mainly investigated with each monomer separately, although mixtures of TEGDMA with either Bis‐GMA or UDMA are used to achieve desired mechanical properties of a restorative material. It is therefore important to consider that combinations of multiple components may result in more or less cytotoxicity or immunomodulation than the individual components would have caused. As demonstrated by Ratanasathien et al., various combinations of monomers present in dentin bonding agents interact to alter the cytotoxicity in vitro (Ratanasathien, Wataha, Hanks, & Dennison, 1995). The purpose of the present study was therefore to investigate how neutrophils responded to the presence of TEGDMA, UDMA, and Bis‐GMA alone or in different combinations in vitro, when it comes to survival and cytokine release.

In the present study, we exposed human neutrophils to different combinations of the methacrylates TEGDMA, Bis‐GMA, and UDMA. An interesting finding was that TEGDMA alone or in combination with Bis‐GMA significantly increased the IL‐8 release from human neutrophils in vitro. IL‐8 is a chemokine with proinflammatory abilities causing recruitment of more neutrophils to the site where it is released, which might result in a prolonged inflammation. We have previously shown the effects of TEGDMA, HEMA, ethyl methacrylate (EMA), and diethylene glycol diacrylate (DEGDA) on the IL‐8 production of human peripheral blood mononuclear cells (PBMCs) in vitro (Alizadehgharib et al., 2017a, 2017b). However, this is the first study to report the ability of TEGDMA alone or in combination to induce IL‐8 secretion by neutrophils.

Neutrophils are programmed to die by apoptosis but this process is delayed or increased depending on the inflammatory milieu. Both microbial factors as LPS or endogenous factors, for example, GM‐CSF, have an antiapoptotic effect on neutrophils, while FASL instead induce apoptosis. We hypothesized that foreign chemicals such as methacrylates also might affect the apoptotic process. In the present study, we used flow cytometry to measure the viability of the neutrophils. The cells were stained with annexin V and 7‐amino‐actinomycin D (7‐AAD), which enables the separation of viable, early apoptotic and late apoptotic/necrotic cells. We could however not see any significant differences in apoptosis (or necrosis) in the presence of the methacrylates, either alone or in combination.

NETosis, that is, extracellular release of DNA associated with histones and granular proteins, has been described as a way for neutrophils to entangle microbes (Guimaraes‐Costa et al., 2012). As reviewed by Guimarães‐Costa et al., NETs can be formed upon exposure to microbes and microbial products (e.g., LPS), but also to cytokines (IL‐8) or synthetic chemicals (Guimaraes‐Costa et al., 2012). In the present study, we show that neutrophils exposed to the methacrylates TEGDMA, UDMA, or Bis‐GMA, alone or in combinations, underwent NETosis to a higher extent than unstimulated cells. Neutrophils are very potent phagocytes and as such they are activated by and engulf microbes as well as various particles regarded as danger signals. Although phagocytes are ductile and can engulf large particles, the phagocytosis process sometimes are unsuccessful, for example, due to overwhelming size and shape of the prey resulting in NETosis with release of intracellular content to the extracellular milieu. The methacrylate monomers are big in size, and one explanation to the NETosis seen in our study might be unsuccessful phagocytosis of the methacrylate particles.

The objective of this study was to add novel knowledge about how neutrophils are affected by exposure to TEGDMA, UDMA, and Bis‐GMA alone or in combinations. Taken all the results from the present study in consideration, it seems that the combination of TEGDMA and Bis‐GMA had a synergistic proinflammatory effect on neutrophils. This is a significant finding since the most commonly used monomers in dental composites are Bis‐GMA and UDMA along with low molecular weight diluents, such as TEGDMA. The combined elution of TEGDMA and Bis‐GMA monomers from poorly cured composite is common and these monomers may come in contact with the local neutrophils in the oral cavity. Since, both IL‐8 release and NETosis are proinflammatory events, it will cause alterations in the normal function of the immune system. This underlines the importance of taking the synergetic interactions in consideration when characterizing effects of chemicals on the immune system.

As the role of methacrylic‐based polymers in dentistry continues to expand, including in fixed, removable, and maxillofacial prosthodontics, orthodontics, preventive dentistry, and operative dentistry, it is important to investigate the biological consequences of these materials. The present findings may help to formulate the least harmful combination of methacrylates and the lowest biological consequences to use in dental materials. However, in order to be able to choose an, from an immunological view, optimal combination of methacrylates to recommend for usage in the clinics, further studies are required on neutrophils as well as on other white blood cell populations.

## CONFLICT OF INTEREST

The authors declare that they have no conflict of interest.

## ETHICS APPROVAL AND CONSENT TO PARTICIPATE

The peripheral blood cells obtained from Sahlgrenska University Hospital blood bank are deidentified, and according to the Swedish legislation section code 4§ 3 p SFS2003:460, no informed consent is needed.
